# Alteration of chromatin high‐order conformation associated with oxaliplatin resistance acquisition in colorectal cancer cells

**DOI:** 10.1002/EXP.20220136

**Published:** 2023-05-29

**Authors:** Peilong Li, Xueying Shang, Qinlian Jiao, Qi Mi, Mengqian Zhu, Yidan Ren, Juan Li, Li Li, Jin Liu, Chuanxin Wang, Yi Shi, Yunshan Wang, Lutao Du

**Affiliations:** ^1^ Department of Clinical Laboratory The Second Hospital of Shandong University Jinan Shandong China; ^2^ Key Laboratory of Systems Biomedicine, Shanghai Center for Systems Biomedicine Shanghai Jiao Tong University Shanghai China; ^3^ Shandong Quality Inspection Center for Medical Devices Jinan Shandong China; ^4^ Wuhan GeneCreate Biological Engineering Co., Ltd Wuhan Hubei China; ^5^ Bio‐X Institutes, Key Laboratory for the Genetics of Developmental and Neuropsychiatric Disorders Shanghai Jiao Tong University Shanghai China; ^6^ Shanghai Key Laboratory of Psychotic Disorders, and Brain Science and Technology Research Center Shanghai Jiao Tong University Shanghai China; ^7^ School of Information Technologies University of Sydney Sydney New South Wales Australia

**Keywords:** 3D spatial structure, colorectal cancer, multi‐omics, oxaliplatin resistance

## Abstract

Oxaliplatin is a first‐line chemotherapy drug widely adopted in colorectal cancer (CRC) treatment. However, a large proportion of patients tend to become resistant to oxaliplatin, causing chemotherapy to fail. At present, researches on oxaliplatin resistance mainly focus on the genetic and epigenetic alterations during cancer evolution, while the characteristics of high‐order three‐dimensional (3D) conformation of genome are yet to be explored. In order to investigate the chromatin conformation alteration during oxaliplatin resistance, we performed multi‐omics study by combining DLO Hi‐C, ChIP‐seq as well as RNA‐seq technologies on the established oxaliplatin‐resistant cell line HCT116‐OxR, as well as the control cell line HCT116. The results indicate that 19.33% of the genome regions have A/B compartments transformation after drug resistance, further analysis of the genes converted by A/B compartments reveals that the acquisition of oxaliplatin resistance in tumor cells is related to the reduction of reactive oxygen species and enhanced metastatic capacity. Our research reveals the spatial chromatin structural difference between CRC cells and oxaliplatin resistant cells based on the DLO Hi‐C and other epigenetic omics experiments. More importantly, we provide potential targets for oxaliplatin‐resistant cancer treatment and a new way to investigate drug resistance behavior under the perspective of 3D genome alteration.

## Graphical abstract

Our research reveals the spatial chromatin structural difference between colorectal cancer (CRC) cells and oxaliplatin resistant cells based on the DLO Hi‐C and other epigenetic omics experiments. More importantly, we provide a series of A/B compartment switching genes as potential targets for oxaliplatin‐resistant cancer treatment and find a new way to investigate drug resistance behavior under the perspective of 3D genome alteration.

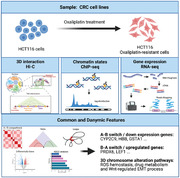

## 1 INTRODUCTION

Colorectal cancer (CRC) is one of the most commonly known malignant tumors with high mortality rate.^[^ ^1^ ^]^ For patients at late recurrence and metastasis stage, chemotherapy has become the most important treatment. Oxaliplatin, the third generation of platinum‐based chemotherapy drug, plays a role as alkylation, causing cross‐linking within and between DNA strands, thereby inhibiting the synthesis of DNA, RNA, and proteins, and activating programmed cell death.^[^ ^2^ ^]^ Applying oxaliplatin is an important therapy especially in metastatic CRC that is ineffective in the treatment of cisplatin and carboplatin. Unfortunately, many patients who undertook several effective chemotherapies, eventually became resistant to oxaliplatin, leading to chemotherapy failure.^[^ ^3^ ^]^ Therefore, it is important to investigate the underlying mechanism of this phenomenon to overcome it and sort for a better anti‐cancer approach for CRC.

At present, the existing studies of oxaliplatin resistance only focus on genome of linear scale,^[^ ^4^ ^]^ and have yet paid attention to the alteration of three‐dimensional (3D) spatial structure of the genome. As eukaryotic DNA is stored in the nucleus by highly folding and condensing it into chromatin, chromatin has complicated but ordered hierarchical conformation in space, which plays a very important role in the process of gene transcription and regulation.^[^ ^5^, ^6^ ^]^ Importantly, the higher‐order chromatin structure is usually disturbed in tumors of different types or other pathological conditions.^[^ ^7^ ^]^ Therefore, understanding the spatial organization of chromatin, such as the spatial distribution of genomes, the way of mutual contact and aggregation, the distribution of regulatory elements, and the structural dynamics of chromatin, should be considered as an important perspective in further recognizing the complex behavior of drug resistance of tumor cells.

The development of Hi‐C technology has made it possible to capture the complex 3D chromatin conformation in the whole genome scale.^[^ ^8^ ^]^ It was revealed that regions of similar epigenomic status tend to contact each other to form A/B compartments composed of large amounts of “active” and “inactive” chromatin regions.^[^ ^9^ ^]^ Chromatin is organized into topologically associated domains (TADs), which is characterized by preferential interaction between loci within the same TAD and isolation from loci in adjacent TADs.^[^ ^10^ ^]^ The area folded into a ring in the TAD is called the chromatin loop, a large part of the chromatin loops are promoter‐enhancer loops, which enable target gene promoter and enhancer that are far apart in the gene sequence to be approached in chromatin space.^[^ ^11^ ^]^ In general, different scales of chromatin structure include A/B compartments, TAD, and loop, in a hierarchical fashion.

In this study, we focus on the alteration of genome 3D structure of CRC when acquiring oxaliplatin resistance. Specifically, we used concentration‐gradient method to establish oxaliplatin‐resistant CRC cell line HCT116‐OxR, conducted advanced DLO Hi‐C experiments to analyze HCT116 and HCT116‐OxR cell lines in A/B compartment, TAD, and loop changes, and performed RNA‐seq and ChIP‐seq omics experiments to investigate the mechanism of CRC regulation in acquired oxaliplatin resistance.

## 2 RESULTS AND DISCUSSION

### 2.1 Conformation of 3D genome in oxaliplatin resistant cells

Oxaliplatin‐resistant CRC cells, named HCT116‐OxR, were established via a concentration‐gradient method in vitro and in vivo (Figure S1A). Cell viability testing showed the cells became resistant to oxaliplatin (Figure S1B,C). Compared to parental HCT116 cells, drug‐resistant cells acquired a more fibroblast‐like appearance, including the loss of cell adherent junctions, tight junctions, and apical‐basal polarity (Figure S1D). In addition, we also verified that the established HCT116‐OxR cell is a subclone of HCT116 cell line by performing STR analysis, which suggests that the differential characteristics in HCT116‐OxR cells were caused by oxaliplatin resistance rather than cell identities.

**FIGURE 1:**
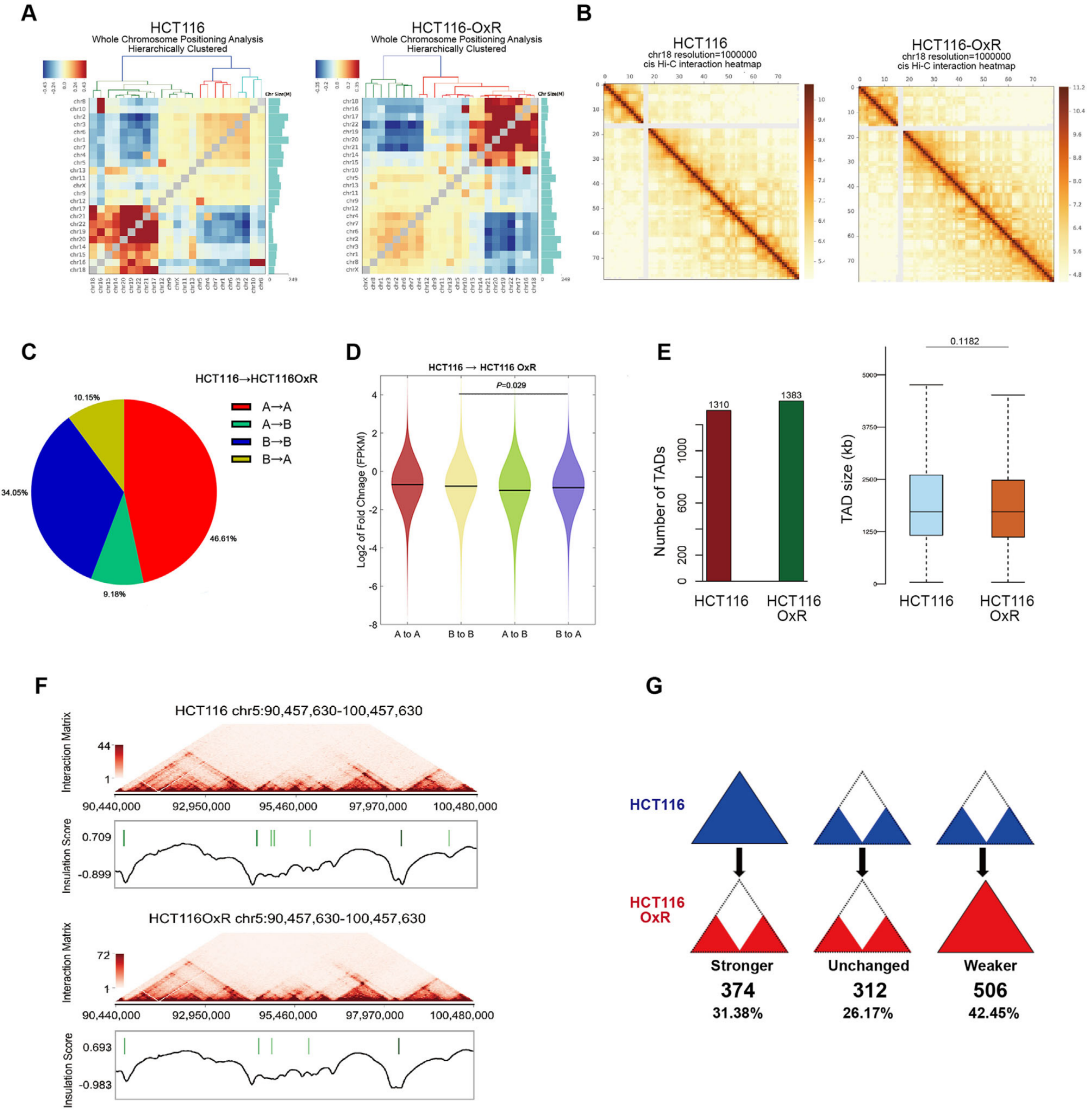
Chromosome conformation changes in colorectal cancer (CRC)‐resistant cells. (A) Inter‐chromosomal clustering heatmap of HCT116 (left) and HCT116‐OxR (left) cells. (B) Comparison of interaction heatmaps of chromosome 5 from HCT116 (left) and HCT116‐OxR cells (right). (C) During HCT116 to HCT116‐OxR, about 19.33% of the genome occurs A/B compartment switching. Regions switching A/B compartment divided by two transitions, A‐B (9.18%) and B‐A (10.15%). (D) Fold change (HCT116‐OxR/HCT116 log2) of mRNA expression (FPKM) of the genes residing at regions with compartment switching. (E) The number and size of topologically associated domains (TADs) were calculated and shown in the two groups of cells. (F) TADs are similar in chromosome 5 between HCT116 and HCT116‐OxR. (G) The strength and weakness of the TAD boundary during drug resistance.

DLO Hi‐C libraries were constructed to identify genome‐wide chromatin structure alteration in oxaliplatin resistance. The genome‐wide interaction data was shown as a heat map of intra and inter‐chromosomal interactions (Figure 1A and Figure S2A). The heat map revealed large‐scale genome distribution in two aspects which agreed with previous knowledge. First, it is consistent with the concept of the chromosome territory^[^ ^12^ ^]^ that chromosome interactions (visualized as gray areas along the diagonal) were more frequent than interactions between chromosomes. Second, the intensity of interaction between chromosomes HCT116 and HCT116‐OxR cells was visually different, indicating that chromosomes were not randomly distributed in the nucleus. In addition, two distinct translocations, t(5;12), t(8;16), were identified. These two translocations have been reported in HCT116 cell line,^[^ ^13^, ^14^ ^]^ but disappear in HCT116‐OxR cells in our study. This indicates that these two translocations may be closely associated with oxaliplatin resistance formation, and HCT116 cells containing these two translocations maybe killed while other cells survived during the formation of oxaliplatin resistance. Therefore, the cells without either translocation could survive the oxaliplatin treatment and develop into resistant cell subline. Finally, chromosome‐wide contact maps showed the expected checkerboard pattern, indicating local associations (TADs) and remote compartmentalization (A/B compartments) (Figure 1B and Figure S2B).

We calculated the first principal component (PC1) from the contact matrix, and further separated the chromatin into A/B compartments at a resolution of 500 kb, reflecting the active and inhibitory chromatin regions respectively (Figure S3A). Overall, 19.33% of the genome switched compartment belongings during drug resistance, of which 9.18% of the genome was converted from compartment A to B, and 10.15% of the genome was converted from compartment B to A (Figure 1C). Moreover, the switched compartments were visibly correlated with gene expression changes. Genes located in A to B‐changed regions in oxaliplatin resistant cells were downregulated while genes located in B to A‐changed regions were upregulated (Figure 1D). Moreover, the median level of gene dysregulation was below 0, indicating the overall downregulation during the formation of resistance (Figure 1D). These data indicated that the A/B compartment belongings can be dynamically flipped in the process of drug resistance. TAD is the basic unit of chromatin conformational folding and function of gene expression regulation. These units are relatively independent of each other, separated by TAD boundaries.^[^ ^15^ ^]^ Insulation algorithm^[^ ^16^ ^]^ was used to determine the TAD boundary by calculating the genome‐wide interaction map with a resolution of 40 kb. Chromosomal TADs were not significantly different in number and size between the two groups (Figure 1E), nor did the TAD structure show significant genome‐wide TAD changes (Figure 1F and Figure S3B). Nevertheless, 42.45% of the TAD boundary of HCT116‐OxR was obviously weakened while 31.38% became stronger (Figure 1G), indicating that the distribution map of TAD boundary in HCT116‐OxR cells showed specific and distinct features compared to HCT116 cells, providing insights to the oxaliplatin resistance nature of HCT116‐OxR.

### 2.2 Peak signal of histone marks and border strength across A/B compartment

**FIGURE 2:**
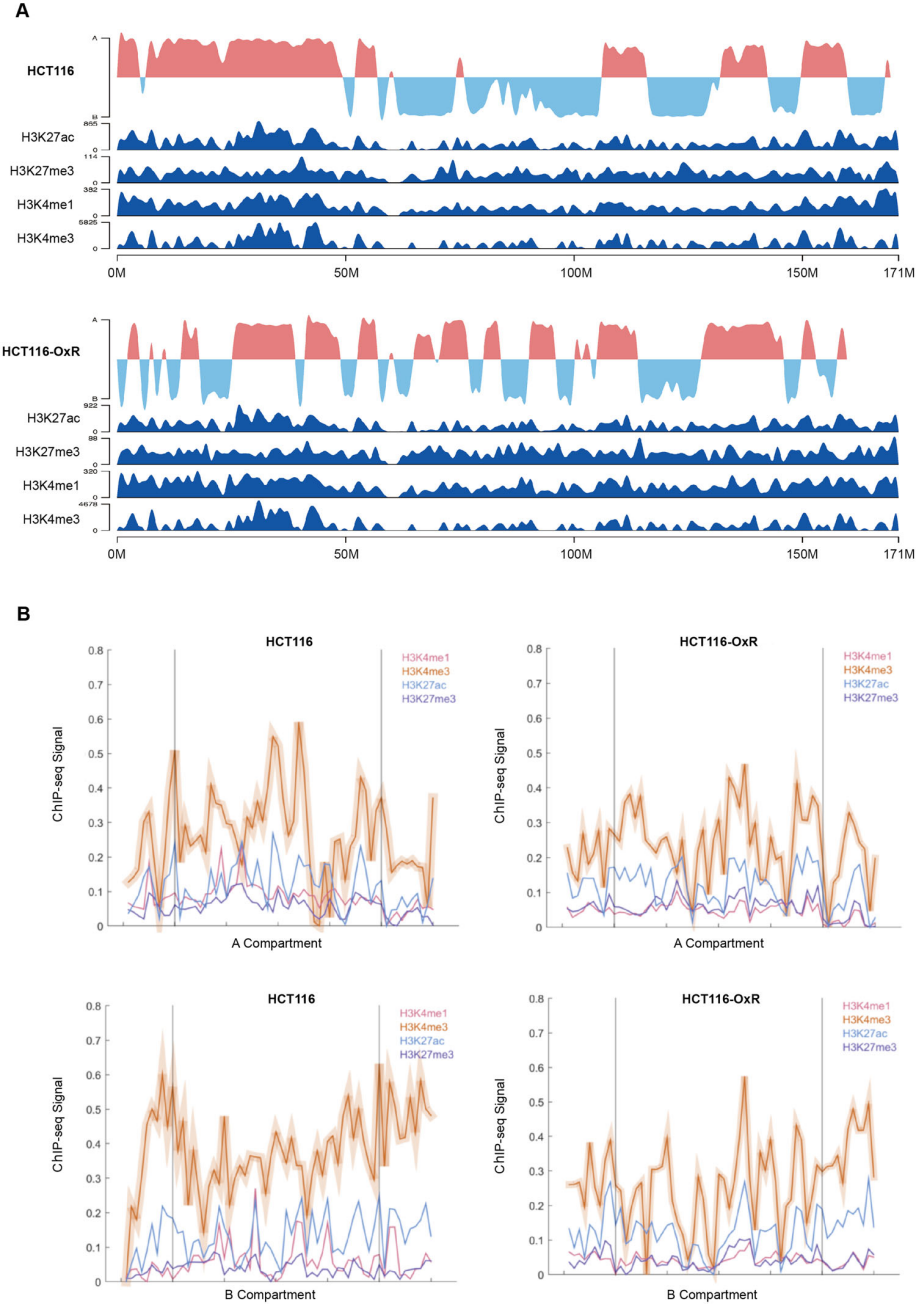
Peak signal of histone marks and border strength across A/B compartment. (A) The active chromosomes markers H3K4me1, H3K4me3, and H3K27ac modification increased with B‐A, and the suppressor chromosome marker H3K27me3 enhanced with A‐B in chromosome 6. (B) For the whole genome, the distribution of four histone markers in compartments A and B were mapped and presented, and the results showed no significant association between compartments and histone modifications.

Specific histone modifications are associated with specific gene activation or suppression states and affect gene regulation in a batch mode. We used ChIP‐seq technology to detect the enrichment of active marks (H3K27ac, H3K4me1, and H3K4me3) and repressive marks (H3K27me3) in HCT116 and HCT116‐OxR cells. The heatmap showed that there were salient differences in chromatin modifications between the two groups, with HCT116‐OxR enriching more H3K27me3 and less H3K4me1 (Figure S4A). This is consistent with the overall downregulation of genes in HCT116‐OxR cells as indicated in Figure 1D, suggesting the histone modifications may be closely correlated with the gene dysregulation mode in drug resistance. The above data also provide clues for further in‐depth study of the effect of histone enrichment along with chromatin on the regulation of tumor cell resistance. By analyzing the signal distribution between the four‐histone marks enrichment and A/B compartment, compartment A was accompanied by transcriptional activation; on the contrary, the transcriptional activation of compartment B was relatively low in chromosome 6 (Figure 2A). However, when we analyze the overall distribution of enrichment in A/B compartment, the association was without statistical significance, suggesting that specific region transcription may play pivotal effects in oxaliplatin resistance, such as chromosome 6 (Figure 2B and Figure S4B).

### 2.3 Correlation between genes dysregulation and A/B compartment switches

**FIGURE 3:**
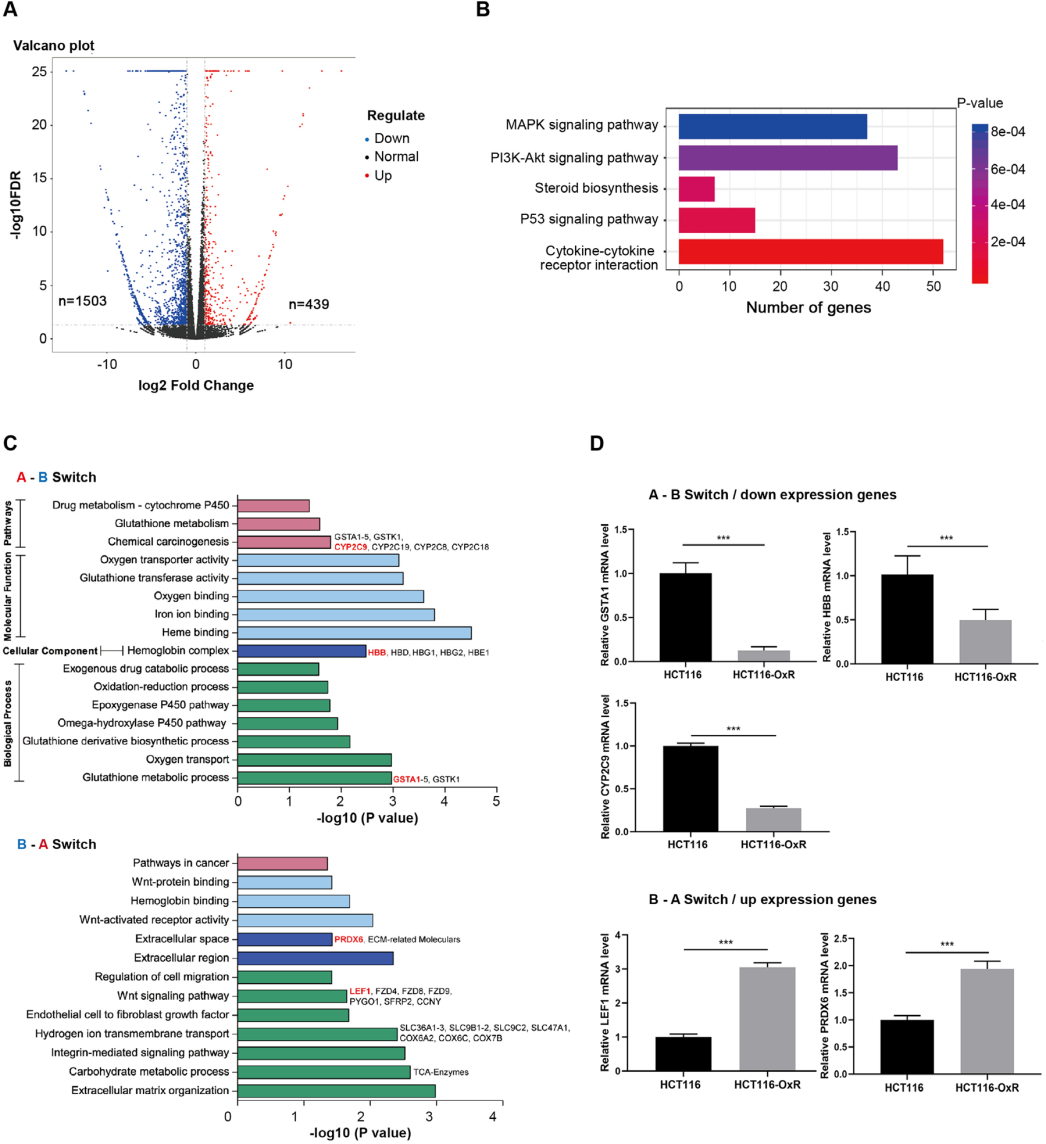
A/B compartment switching leads to genes dysregulation respond to oxaliplatin. (A) The volcano map shows the overall distribution of differential genes, and genes with significant differential expression are marked with red dots (up‐regulated) and blue color dots (down‐regulated) indicate that genes with no significant differential expression are indicated by black dots. *q* < 0.05, | log2fold change | > 1. A total of 1503 and 439 genes were identified as lower and higher expressed in HCT116‐OxR cells compared to HCT116 cells. (B) Enrichment analysis of KEGG pathway of differentially expressed genes. (C) Enrichment analysis of A‐B / down‐regulated genes and B‐A/up‐regulated genes respectively. (D) qRT‐PCR experiments verified the expression of key genes GSTA1, hemoglobin subunit β (HBB), CYP2C9, LEF1, and PRDX6, ^***^ *P* < 0.001.

RNA‐seq was performed to further characterize the differences of gene expression between HCT116 and HCT116‐OxR cells. A total of 439 up‐regulated genes and 1503 down regulated genes of HCT116‐OxR were identified (Figure 3A and Figure S5A,B). To verify the results, we performed quantitative real‐time PCR (qRT‐PCR) on 6 most regulated genes, and the results showed high reproducibility with RNA‐seq (Figure S5C). KEGG enrichment analysis showed that the differential genes were mainly enriched in the MAPK signaling pathway, PI3K‐Akt signaling pathway, p53 signaling pathway, and other classic drug resistance signaling pathways (Figure 3B).

Since oxaliplatin resistance in HCT116 cells was related to chromatin conformation alteration, especially at the compartment scale, we sought to reveal the specific regulatory mechanism from the perspective of compartment switching. Enrichment analysis was performed on the “A to B” (A‐B) and “B to A” (B‐A) switched genes, respectively. The results showed that A‐B switched genes were mainly enriched in the pathways of glutathione (GSH) metabolic process, drug metabolism, and oxygen transport related processes. Those B‐A switched genes were significantly enriched in the pathways of cell migration related processes, including WNT signaling pathway and extracellular matrix (ECM) terms, as well as energy metabolism, for example, carbohydrate metabolic process (focused on tricarboxylic acid cycle) and electron transport chain responsible for mitochondrial oxidative phosphorylation (Figure 3C).

To verify the expression changes in compartment switched genes, we detected expression of five pivotal genes that engaged in the enriched terms and pathways, including GSTA1, the member of GSH transferases (GST) subfamily,^[^ ^17^ ^]^ a crucial enzyme in drug metabolism CYP2C,^[^ ^18^ ^]^ HBB (hemoglobin subunit β),^[^ ^19^ ^]^ LEF1 (lymphoid enhancer binding factor 1), which is involved in WNT signaling pathway for target activation,^[^ ^20^ ^]^ and PRDX6, an important peroxidase for maintaining reactive oxygen species (ROS) homeostasis.^[^ ^21^ ^]^ The qRT‐PCR results showed that the A‐B switched genes were inhibited, and B‐A switched genes were significantly up‐regulated in HCT116‐OxR cells (Figure 3D). Together, it is explicit that the sensitivity to oxaliplatin is impaired by compartment switching‐induced dysregulation in HCT116 cells.

### 2.4 Oxaliplatin resistance is related to reduced ROS and impeded drug metabolism

**FIGURE 4:**
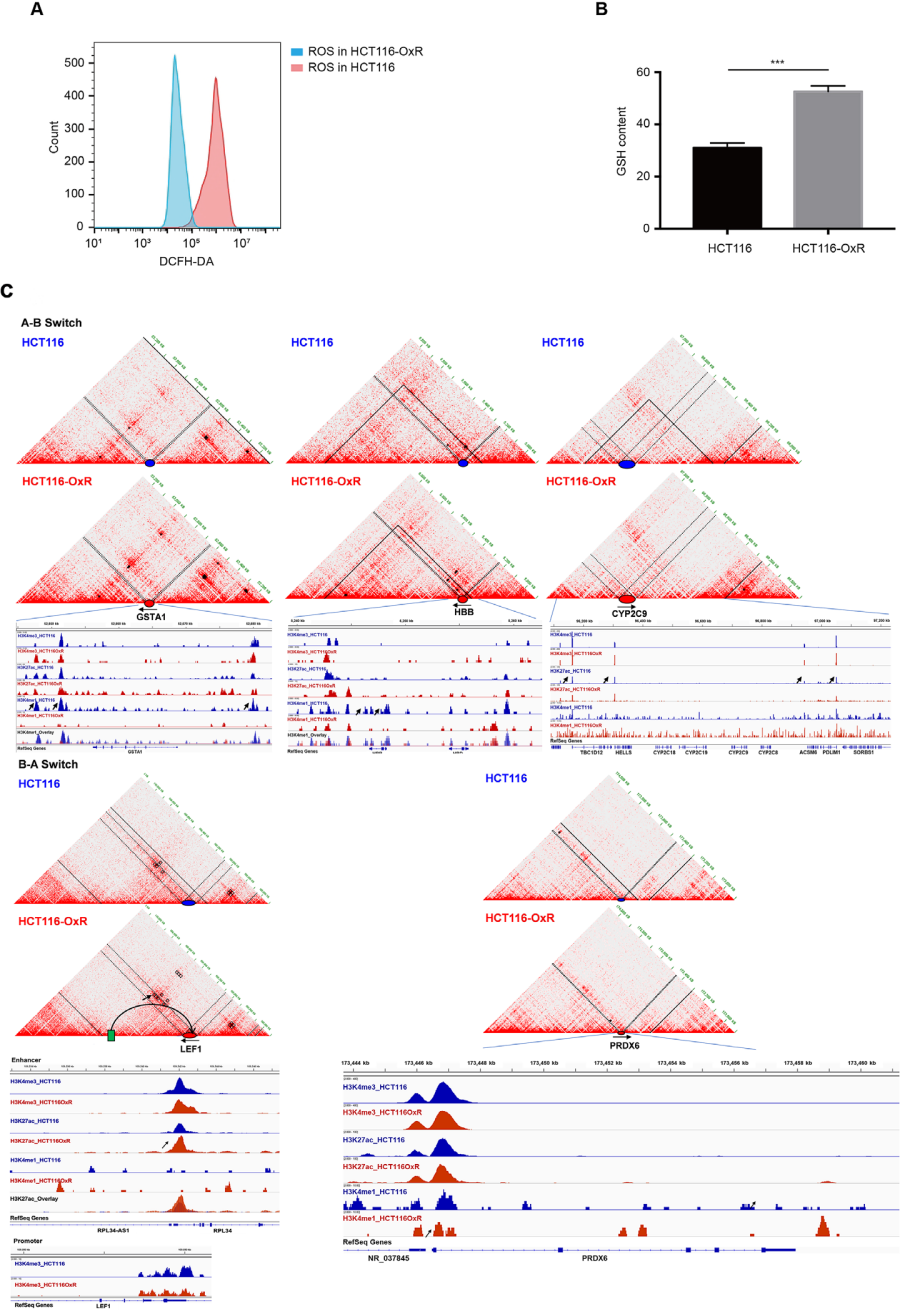
Oxaliplatin resistance is owing to the reduced reactive oxygen species (ROS) and impeded drug metabolism. (A) The level of ROS in the HCT116 and HCT116‐OxR cells. (B) The content of glutathione (GSH) in the HCT116 and HCT116‐OxR cells, ^***^ *P* < 0.001. (C) In‐depth analysis of the TAD, loop, and modification levels of histone markers of 5 key genes. TAD borders labeled in solid black lines, and the genomic coordinates of targets, which were colored in blue and red in HCT116 and HCT116‐OxR cells respectively, pointed by the double dashed lines. The green bar indicating the potential enhancer that interacts with the promoter of LEF1 gene. Arrows indicate the differential enriched regions.

A hypothesis was thus proposed that compartment‐controlled genes significantly enriched in GSH metabolic process, hemoglobin, and peroxidase might lower intracellular ROS levels. Concretely, it has been well‐known that the activated 1‐cys peroxidase PRDX6 can effectively remove peroxides and thereby reducing the level of intracellular ROS.^[^ ^22^ ^]^ Meanwhile, the oxidized Cp residue could be restored by GSH,^[^ ^21^ ^]^ the silenced GSTA class molecules can theoretically accumulate high levels GSH that facilitate PRDX6 reaction loop, so as to further reduce the intracellular ROS level.^[^ ^23^ ^]^ In addition, the inhibition of hemoglobin subunits with A‐B switching induces an intracellular hypoxic environment, which renders HCT116 cells apt to glycolysis and lowers ROS levels.^[^ ^24^ ^]^ To verify this hypothesis, one needs to answer whether intracellular ROS and GSH were affected in HCT116‐OxR cells, and what is the regulatory mechanism during compartment switching. We next examined changes in ROS and GSH levels by flow cytometry, the results showed that intracellular ROS were significantly decreased in oxaliplatin‐resistant cells as indicated by the fluorescence signals of 2′,7′‐dichlorofluorescin diacetate (DCFH‐DA) (Figure 4A). Furthermore, accumulation of GSH was detected in HCT116‐OxR cells compared with HCT116 (Figure 4B). Notably, GSH has been previously reported as an intracellular antidote to neutralize oxaliplatin.^[^ ^25^ ^]^ In addition, the expression level of CYP2C9 has been verified to be significantly down‐regulated in drug resistant cells, manifesting that impeded drug metabolism might be another culprit for oxaliplatin‐resistance in cancer cells. Together, it can be concluded that drug resistance to oxaliplatin attributes to ROS reduction and impeded drug metabolism in HCT116 cells.

To explore the regulatory mechanism of ROS homeostasis mediated by compartment switching, we analyzed the TAD and loop positioning in combining the histone marks recruitment in candidates. As shown in Figure 4C, the enriched peaks of H3K4me1 and H3K4me3 at regulatory regions of GSTA1 were significantly reduced in drug resistant cells. On the contrary, the peak signals of enhancer marks H3K4me1 and H3K27ac were obviously increased in HCT116‐OxR cells accompanied by TAD borders weakened in peroxidase PRDX6 (Figure 4C and Figure S3C). With regard to hemoglobin subunit HBB for hypoxic signals and drug metabolic enzyme CYP2C9 in switched compartment B, similarly to GSTA1, the modification levels of active modifications H3K4me1 and H3K27ac were significantly decreased in HCT116‐OxR cells. Specially, a new loop was formed in LEF1 gene area, and a significantly elevated signal of H3K27ac was observed in HCT116‐OxR cells compared to that in the control, suggesting the novel enhancer‐promoter loop in LEF1 gene maybe involved in the drug resistance (Figure 4C).

### 2.5 Compartment switching genes can affect cellular response to oxaliplatin by regulating ROS homeostasis

To further explore the relationship among compartment switching genes, ROS homeostasis, and oxaliplatin resistance. We overexpressed A‐B switching genes, CYP2C9 and GSTA1 in HCT116‐OxR cells (Figure S6A) and examined intracellular changes of ROS by flow cytometry. The results showed that, compared with the control group, both CYP2C9 and GSTA1 overexpressing cells could up‐regulate ROS levels in HCT116‐OxR (Figure S6B). We further detected the responsiveness of CYP2C9 and GSTA1 overexpressing cell lines to oxaliplatin by CCK8 and flow cytometry. Interestingly, HCT116‐OxR cells overexpressed with CYP2C9 and GSTA1 were more sensitive to oxaliplatin compared with the resistant cell lines, with decreased IC50, and increased proportion of apoptotic cells (Figure S6C,D). At the same time, we also knocked down the B‐A switching gene, PRDX6, in HCT116‐OxR cells, and detected the level of intracellular ROS and the response to oxaliplatin. The knockdown efficiency of si‐PRDX6 could reach 0.2–0.3 detected by qRT‐PCR and Western Blot (Figure S6E). The PRDX6 knockdown cell line had increased ROS levels (Figure S6F) and increased responsiveness to oxaliplatin compared with control cells (Figure S6G,H). The above results demonstrated that compartment switching genes can affect cellular response to oxaliplatin by regulating ROS homeostasis, and that oxaliplatin resistance is the result of a multi‐gene interaction.

### 2.6 B‐A switched LEF1 promotes cell migration and invasion antagonistic to oxaliplatin

Since we have observed an enhanced EMT and invasive ability of HCT116‐OxR cells, we sought to explore whether chromatin structure alteration was engaged in the cell migration and invasion processes. The results showed that genes in B‐A switched compartments had an obvious enrichment in ECM‐related terms and WNT pathway, indicating that cell morphology and motility were modulated by compartment switching in HCT116‐OxR cells (Figure 3C).

As the crucial transcription factor for target activation in WNT/β‐catenin pathway, LEF1 was selected to further investigate the regulatory pattern. As shown in Figure 4C, Hi‐C matrices displayed an emerging loop in drug‐resistant cells. We knockdown LEF1 expression in drug‐resistant cells (Figure S7A), and observed that the migration and invasion ability of drug‐resistant cells was reduced (Figure S7B), and the EMT characteristics were weakened (Figure S7C). In addition, LEF1 knockdown cells have increased responsiveness to oxaliplatin (Figure S7D). Together, these findings indicated that compartment switching was involved in oxaliplatin resistance‐induced EMT process in HCT116‐OxR cells.

In this study, we employed DLO Hi‐C technology to characterize the chromatin structure and give a comprehensive overview of the spatial structure of CRC cells before and after resistance‐oxaliplatin (Figure 1; Figures S2 and S3). Then, RNA‐seq and ChIP‐seq techniques were applied to detect changes in gene expression and histone modifications in CRC cells in the process of acquiring oxaliplatin resistance, respectively (Figures 2 and 3). We found the differential genes were enriched in classical drug resistance pathways (Figure 3B), combined with A/B compartment analysis of differential genes, a large number of down‐regulated genes were converted from A to B; the transformation from B to A was accompanied by the enhancement of the intensity of H3K4me1, H3K4me3, and H3K27ac modified active chromosomes (Figure 3C). We performed a series of functional assays on candidate A/B compartment transformation genes and then inferred reduction of ROS in cancer cells, inhibition of drug metabolism, and enhanced metastatic capacity through chromatin conformation changes were responsible for oxaliplatin resistance (Figures S6 and S7). The above results confirmed that the genome 3D conformation was abnormally altered during oxaliplatin‐resistance in CRC cells, providing GSTA1, PRDX6, LEF1 as potential therapeutic targets. However, we need to state the limitation of this study that detailed regulatory mechanism and relationships between genome alteration and oxaliplatin resistance need to be explored in more in‐depth studies as future works.

## 3 CONCLUSION

In this study, we performed multi‐omics study by combining DLO Hi‐C experiments as well as other genomics experiments to explore the epigenetic landscapes. These omics data provide resources for exploring genome‐wide 3D spatial structure evolution during CRC oxaliplatin resistance and for gaining insights into the functional role of chromatin structure in tumorigenesis and resistance development. Using these data, we can mine the structure of any genomic locus, which will open up new ideas for studying drug resistance in tumor cells.

## 4 EXPERIMENTAL SECTION

### 4.1 Cell lines

CRC cell HCT116 was purchased from Cell bank of the Chinese Academy of Sciences (Shanghai, China). Oxaliplatin (MedChemExpress) was used to establish Oxaliplatin‐resistant cell line (HCT116‐OxR) as previously described.^[^ ^26^, ^27^ ^]^ Cells were cultured in RPMI1640 supplemented with 10% fetal bovine serum (FBS) at 37°C in 5% CO_2_ and 95% air.

### 4.2 Transfection

The pcDNA3.1(+) vector, CYP2C9, and GSTA1 overexpression plasmid, siRNAs duplexes for silencing PRDX6 and LEF1 were generated by GenePharma (Shanghai, China). The transfection process was operated by using Lipofectamine 3000 (Invitrogen) as per the product guidelines. Related sequences for siRNAs are shown below:

**Table:** 

Si‐NC	sense: UUCUCCGAACGUGUCACGUTT
	antisense: ACGUGACACGUUCGGAGAATT
Si‐PRDX6	sense: GGAACUUUGAUGAGAUUCUTT
	antisense: AGAAUCUCAUCAAAGUUCCTT
si‐LEF1	sense: CCGUGAAGAGCAGGCUAAATT
	antisense: UUUAGCCUGCUCUUCACGGTT

### 4.3 Apoptosis assay

Annexin V FITC/PI double staining assay kit (Bestbio, ShangHai, China) was used for the detection of cell apoptosis induced by oxaliplatin. Briefly, HCT116‐OxR cells were transfected as indicated, 12 h after transfection, cells were treated with 100uM oxaliplatin for 48 h. Cells and supernatant were then collected, apoptosis analysis was performed following the manufacturer's protocol.

### 4.4 Cell migration and invasion assay

Cell migration was measured with Transwell assays, briefly, cancer cells (1 × 10^5^ cells/well) in serum‐free media were placed on each 8.0‐μm pore size membrane inserted in 24‐well plates. RPMI1640 plus 20% FBS was placed in the bottom wells as attractants. After 24 h, cells that did not migrate were removed from the top side of the inserts with a cotton swab. Cells that had migrated to the underside of the inserts were washed with PBS, fixed with methanol, stained with 0.1% crystal violet, washed three times with PBS, and imaged by Inversion Microscope. The invasion assay was done in a similar fashion except the 8.0‐μm pore size membrane inserts were coated with Matrigel.

### 4.5 qRT‐PCR

Total RNA was isolated from cells using TRIzol reagent and subjected to reverse transcription using PrimeScript™ RT reagent kit according to the manufacturer's instructions. The mRNA levels were determined using Takara SYBR Premix Ex Taq II. The following primers were used for qRT‐PCR:

KDM5D: forward 5′‐3′: TCAGTCAAAAAGGGCTCGGA;
reverse 5′‐3′: GGTCTGTGGAAGGTGTCAGG.
EIF1AY forward 5′‐3′: CAGGCGCAGGGGTAAAAATG;
reverse 5′‐3′: ACACAATGCTTCCAATCGTCC.
USP9Y forward 5′‐3′: AATTCCGTAGCAACTCCTCCT;
reverse 5′‐3′: CAAAACAGGAACCACCCATCG.
IFI44L forward 5′‐3′: GACTTCTCAAAGCCGGGTCA;
reverse 5′‐3′: CCTTCATGGGGTCCAGTTCC.
XAF1 forward 5′‐3′: GGGTAAATGTTGTCCAGACTCAG;
reverse 5′‐3′: GCTTGACTTGGAAGAGATGAAGG.
GBP1 forward 5′‐3′: AGTCTTCATCAGGAGTTCCTTCAAA;
reverse 5′‐3′: TTCCCGCCTTCACTTCTTCTT.
GSTA1 forward 5′‐3′: AAAATCGCTACTTCCCTGCCTT;
reverse 5′‐3′: ATAAGACTGGAGTCAAGCTCCTCG.
HBB forward 5′‐3′: GAAGTCTGCCGTTACTGCCC;
reverse 5′‐3′: AGCCTTCACCTTAGGGTTGC.
CYP2C9 forward 5′‐3′: GAAGGAAAAGCACAACCAACCA;
reverse 5′‐3′: TCTGTCCCAGCTCCAAACAA.
LEF1 forward 5′‐3′: AACACCCCGATGACGGAAAG;
reverse 5′‐3′: TGCACCACGGGCACTTTATT.
PRDX6 forward 5′‐3′: GCCACCCCAGTTGATTGGAA;
reverse 5′‐3′: GGTGAAGACTCCTTTCGGGA.
GAPDH: forward 5′‐3′: GCACCGTCAAGGCTGAGAAC;
reverse 5′‐3′: TGGTGAAGACGCCAGTGGA.

### 4.6 Western blotting

Cells were lysed with RIPA lysis buffer containing protease inhibitors. Equal amounts of the extracts were loaded, subjected to 10% SDS‐PAGE, transferred onto a polyvinylidene fluoride (PVDF) membrane. Antibodies against E‐cadherin (3195S), N‐cadherin (13116S), Vimentin (5741S), and β‐actin (8457) were obtained from Cellular Signaling Technology (CST), antibodies against PRDX6 (ab133348) and LEF1 (ab137872) were obtained from Abcam. PVDF membrane was incubated with primary antibody overnight at 4°C. Then, HRP‐conjugated goat anti‐rabbit and sheep anti‐mouse IgG (H+L) (CST) were used as secondary antibodies, and the immunoblots were visualized by using ECL Reagents.

### 4.7 Preparation of Hi‐C libraries

DLO Hi‐C was performed as previously described. HCT116 and HCT116‐OxR cells were first digested by restriction enzymes, then subjected to a series of treatments such as Mmel linker ligation, fragment‐end phosphorylation, in situ proximity ligation, reversal of cross‐linking and purification of the DLO Hi‐C DNA fragments, and finally the Illumina sequencing adaptor was ligated to the DLO Hi‐C DNA fragment to construct sequencing library. In Hi‐C data, there are deviations from different sources, such as fragment length, GC content, and Mapp ability at mega base resolution.^[^ ^28^ ^]^ We used ICE method^[^ ^29^ ^]^ The normalized interaction matrix was used for further analysis. Distribution of restriction sites varied throughout the genome. HiCNorm^[^ ^30^ ^]^ scripts were used to calculate the location of restrictive sites, and BEDtools^[^ ^31^ ^]^ were used to generate upstream and downstream readings with specific lengths. Then, all readings were aligned with the human genome (hg19 source from UCSC) using the BWA package.^[^ ^32^ ^]^ The percentages of unique mapping readings were calculated using readings with a MAPQ quality score ≥ 20. The GEO Accession number is GSE138652.

### 4.8 Eigenvector analysis

A/B compartments were determined as described previously.^[^ ^29^ ^]^ The hic‐lib package was used to compute the eigenvector decomposition of 1‐Mb interaction matrix. The first eigenvector (PC1) was used to define compartments A and B. Bins with a value greater than 0 were considered to be compartment A, while bins with a value less than 0 were considered to be compartment B. Only changes in PC1 values from positive to negative or from negative to positive were considered to represent differential compartments.

### 4.9 TAD boundary calling

The genome was divided into 40‐kb windows. For each window, the frequency of interaction within the upstream and downstream 2 Mb was compared, and the TAD boundary was determined by the directional index as previously described.^[^ ^33^ ^]^ If the area between two adjacent boundaries was less than 400 kb, the area was marked as a TAD boundary. Then, all the intervals between the two samples were determined. If the regions overlapped in different samples, they were considered to be overlapping boundaries.

Detailly, we used the Insulation Score approach to identify the boundaries of the TAD. The algorithm principle is that the genome is first divided into n bins of the same size according to a certain resolution. For each bin, we calculate the total number of interactions between the bin and other bins in the “community” as its “community contacts.” In order to measure the community activity level of the bin in the entire chromosome, the mean of the “community activity” of all bins will be calculated (mean), and calculate the insulation score of the bin by the formula log_2_(community contacts/mean). In order to determine the boundary of the TAD, it is necessary to find a bin with relatively less interaction with the surrounding area and a relatively low degree of activity. In order to determine such a bin, a smaller range (such as 200 kb) is set around this bin. Within the scope, if the insulation score of the upstream bin of the bin continues to decrease to the lowest point of the bin, and the insulation score of the downstream bin continues to increase, then the bin is the candidate TAD boundary (reference point). For further screening, the mean left of the insulation score in the upstream 100 kb range of each bin (mean left) and the mean value of the downstream insulation score (mean right) will be calculated, and the delta of the changed bin will be calculated by the formula delta = mean left—mean right. If the delta value is higher than the set value, the reference point is considered as the TAD boundary.

### 4.10 Loop creation and aggregate peak analysis

The Juicer^[^ ^34^ ^]^ pipeline's HiCCUPS^[^ ^35^ ^]^ was used to detect locally enriched peaks. The hic. format file was created by Juicer with default resolutions of 2.5, 1 Mb, 500, 250, 100, 50, 25, 10, and 5 kb. Then, all locally enriched peaks were determined by HiCCUPS with default parameter resolutions of 5, 10, and 25 Kb.

### 4.11 RNA‐seq and analysis

The RNA‐seq libraries were generated with VAHTS Stranded mRNA‐seq Library Prep Kit for Illumina and the samples were sequenced as 150‐bp paired‐end reads using a sequence instrument. For the RNA‐seq analysis, clean reads were aligned with Bowtie 2.2.1.0 index human genome (hg19). The mapping reads for each sample were assembled using StringTie with default settings. All transcripts from the samples were merged to reconstruct the full transcript group using the Perl script. After producing the final transcriptome, StringTie and DESeq2 1.18.1 were used to estimate the expression level of all transcripts. StringTie was used to calculate FPKM to perform the level of expression of mRNA. Differential gene expression was invoked using DESeq2 1.18.1. To find significant differentially expressed genes, we used *p* < 0.05 and > 1 log_2_ fold change. The enrichment analysis of GO terminology (http://geneontology.org/) was based on hypergeometric testing with a threshold value of *p* < 0.05 to detect significant enrichment of detected genes. We used R‐Package to test differentially expressed genes in KEGG pathway (http://www.kegg.jp/). The GEO Accession number is GSE138647.

### 4.12 ChIP‐seq and analysis

Cells were cross‐linked with 1% formaldehyde at room temperature (RT) for 10 min and quenched with 0.125 m glycine for 5 min. For nuclear extract preparation, the cross‐linked cells were lysed in lysis buffer (50 mm Tris‐HCl, 150 mm NaCl, 1 mm EDTA, 1% Triton X‐100, 0.1% SDS, supplemented with protease inhibitors), incubated on ice for 10 min. We precipitated nuclei by centrifugation at 12,000 rpm. After 15 min of centrifugation, the chromatin was used for H3K27ac(ab4729), H3K4me1(ab8895), H3K4me3(ab8580), or H3K27me3(ab6002) for immunoprecipitation overnight. Immunoprecipitation with unrelated nonspecific immunoglobulins was used as a negative control in each experiment. The antibody‐bound DNA was then washed with a low‐salt washing buffer (1% Triton X‐100, 1 mm EDTA, 50 mm Tris‐HCl, 150 mm NaCl, and a high‐salt washing buffer (1% Triton X‐100, 1 mm EDTA, 50 mm Tris‐HCl, 500 mm NaCl), then elution buffer (1% SDS, 100 mm NaHCO3) twice. ChIPed DNA was reverse‐crosslinked and purified for construction of DNA library, and then sequenced. The library was sequenced on Illumina HiSeq X Ten using 150 bp pair end chemistry. Bowtie2.1.0 (http://bowtie‐bio.sourceforge.net/bowtie2/index.shtml) was used to align reads with the hg19 human genome, excluding those with more than one mismatch or that can be mapped to multiple genome locations. MACS2 2.1.1 was used for peak call (*p*‐value threshold = 10–5) with default parameters. The overlapping enrichment regions were merged and considered as promoters when located within 1000 bp of the annotated transcriptional initiation site (TSS) of hg19, and as presumed distal enhancers when located more than 1000 bp away from TSS. The GEO Accession number is GSE138654.

### 4.13 ROS and GSH detection

The ROS were measured by active oxygen detection kit‐DCFHDA method according to the manufacturer's instructions (Beyotime, S0033S, Shanghai, China). DCF fluorescence was detected by flow cytometry at 488 nm excitation wavelength and 525 nm emission wavelength. Totally 10,000 cells were analyzed per sample. The GSH concentration was measured by GSH/GSSG detection kit according to the manufacturer's instructions (Beyotime, S0053).

### 4.14 Qualification and statistical analysis

All experiments were performed in triplicate. Data conformed to a normal distribution as assumed and were presented as mean ± SD. Comparison between two groups was analyzed using the Student's *t*‐test. The variance was mild within each group of data and similar between the groups that were being statistically compared. Statistical analyses were performed using GraphPad Prism (v8.0.1, GraphPad Software Inc., San Diego, CA, USA).

## CONFLICT OF INTEREST STATEMENT

The authors declare no conflicts of interest.

## Supporting information

Supporting Information

## Data Availability

All data related to this work are present in the article and in the Supporting Information. Any other data associated with this work are openly available in “GEO” at https://www.ncbi.nlm.nih.gov/geo/, the accession number are GSE138652, GSE138647, and GSE138654.
