# Supplementary material for: Alteration of chromatin high‐order conformation associated with oxaliplatin resistance acquisition in colorectal cancer cells
Source: Exploration (Beijing). 2023 May 29;3(4):20220136. doi: 10.1002/EXP.20220136 (PMC10624369; doi:10.1002/EXP.20220136)
Supplement: Supplementary file 1 — Supporting Information [file EXP2-3-20220136-s001.pdf]

Supporting Information

Alteration of chromatin high-order conformation associated with oxaliplatin resistance acquisition in colorectal cancer cells

Peilong Li[1*], Xueying Shang[2*], Qinlian Jiao[3*], Qi Mi[1]*, Mengqian Zhu[1], Yidan Ren[1], Juan Li[1], Li Li[4], Jin Liu[4], Chuanxin Wang[1#], Yi Shi[5,6,7#], Yunshan Wang[1#], Lutao Du[1#]

[1] Department of Clinical Laboratory, The Second Hospital of Shandong University, 247 Beiyuan Street, Jinan 250033, Shandong, China.

[2] Key Laboratory of Systems Biomedicine, Shanghai Center for Systems Biomedicine, Shanghai Jiao Tong University, Shanghai 200240, China.

[3] Shandong Quality Inspection Center for Medical Devices, 15166 Century Avenue, Jinan 250000, Shandong, China.

[4] Wuhan GeneCreate Biological Engineering Co., Ltd. 666 Gaoxin Avenue, Wuhan 430206, China.

[5] Bio-X Institutes, Key Laboratory for the Genetics of Developmental and Neuropsychiatric Disorders, Shanghai Jiao Tong University, 1954 Huashan Road, Shanghai, 200030, China

[6] Shanghai Key Laboratory of Psychotic Disorders, and Brain Science and Technology Research Center, Shanghai Jiao Tong University, 1954 Huashan Road, Shanghai, 200030, China

[7] School of Information Technologies, University of Sydney, Sydney, NSW 2006, Australia

*These authors contributed equally to this work.

# Corresponding Authors: Chuanxin Wang, cxwang@sdu.edu.cn

Yun-shan Wang, wangyunshan135@126.com

Yi Shi, yishi@sjtu.edu.cn

Lu-tao Du, lutaodu@sdu.edu.cn

Supplementary figures

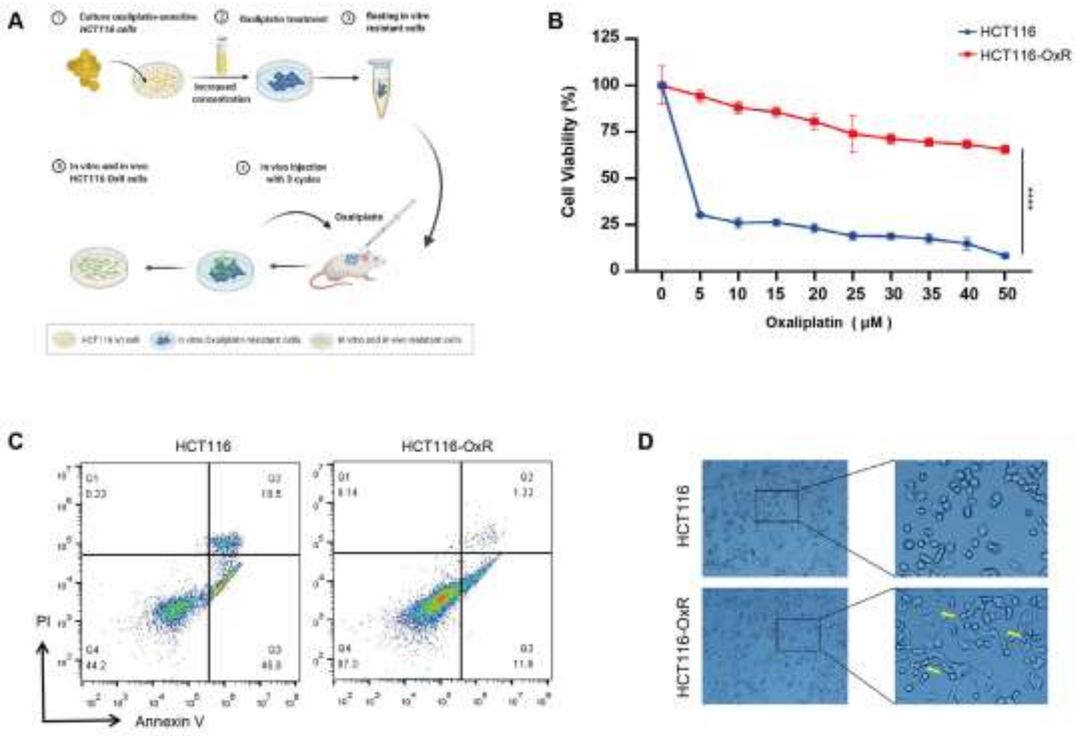

Figure S1. Establishment and validation of oxaliplatin resistant HCT116 cells.
A Construct oxaliplatin-resistant HCT116-OxR cell using concentration gradient method and nude mouse injection method. **B** HCT116-OxR was significantly insensitive to oxaliplatin by CCK8 assay, ***P<0.001. **C** The percentages of live cells (Annexin V−/PI−), and apoptotic cells (Annexin V+/PI− & Annexin V+/PI+) was evaluated among the HCT116 and HCT116-OxR cells pre-treated with oxaliplatin (100 μM, 24 h). **D** Compared with HCT116, the morphology of HCT116-OxR cells became fusiform and the polarity was lost.

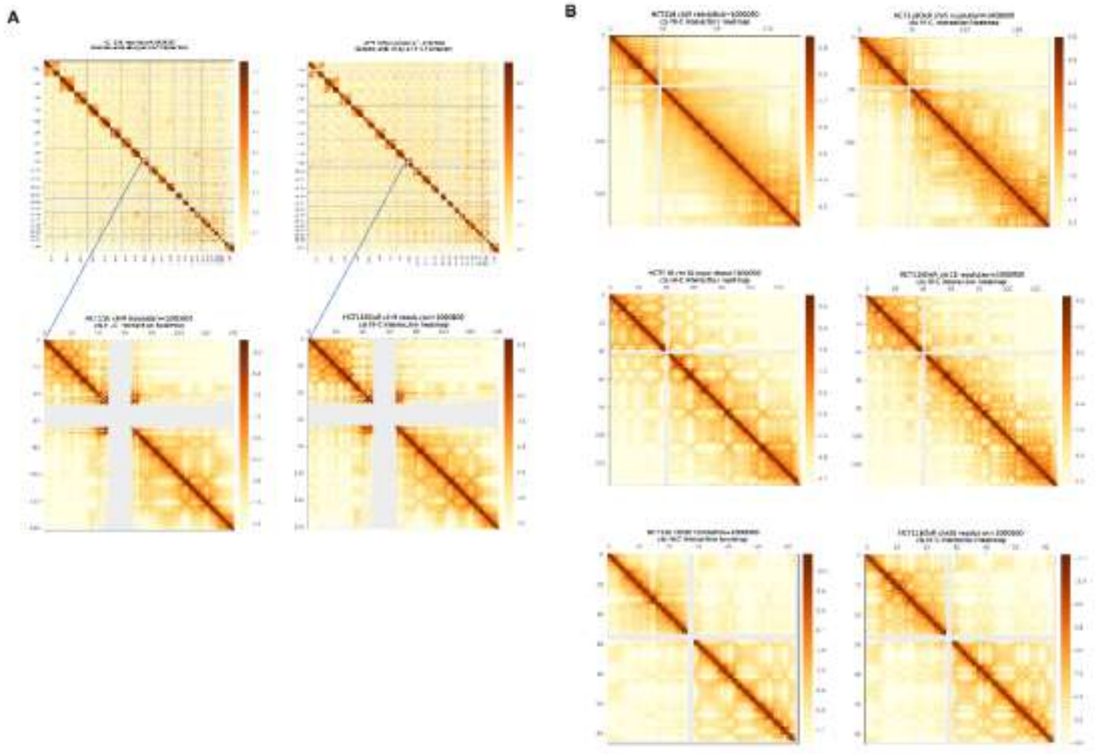

Figure S2. The repeatability chromatin remodeling and merged interaction heatmap in HCT116 and HCT116-OxR cells.

A Whole genome interaction maps show that intrachromosomal interactions are more frequent. **B** The heat map of the interaction of multiple chromosomes between the two groups of cells showed no significant difference.

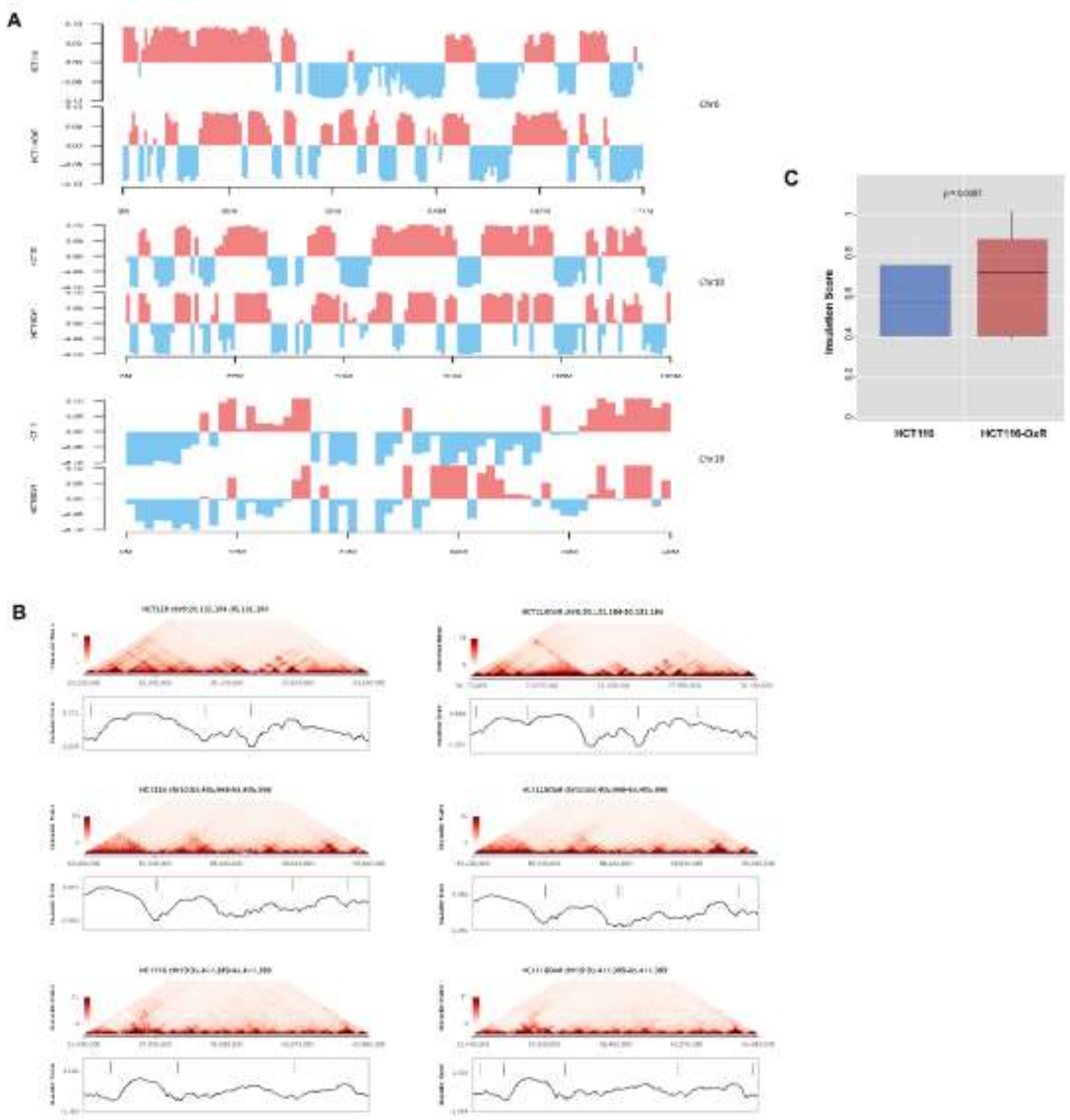

Figure S3. The difference between the A/B area and TAD structure of the two groups of cells.

A In the process of drug resistance, there are more genes in multiple chromosomes with A/B compartment transformation. **B** The structure of TAD is highly conservative during the occurrence of drug resistance. **C** The insulation score comparison between HCT116 and HCT116-OxR cells surrounding the gene PRDX6 at chr 1: 167,800,000–182,840,000. The sliding window was defined as 40 kb, and regions with low score were insulating.

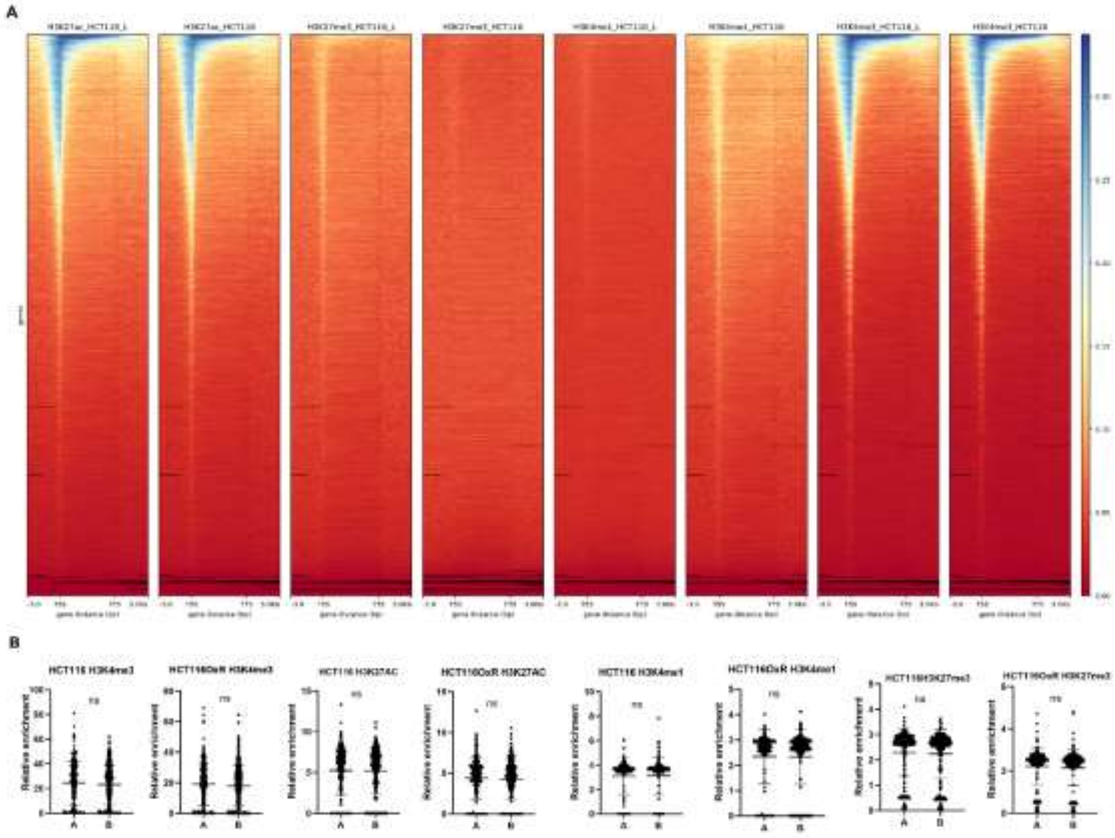

Figure S4. Modification of H3K27ac, H3K4me1, H3K4me3 and H3K27me3 in HCT116 and HCT116-OxR cells.

A H3K27ac, H3K4me1, H3K4me3 and H3K27me3 modified gene distribution in two cells. **B** The relative enrichment of indicative histones in A or B compartments (%) in HCT116 and HCT116OxR cells.

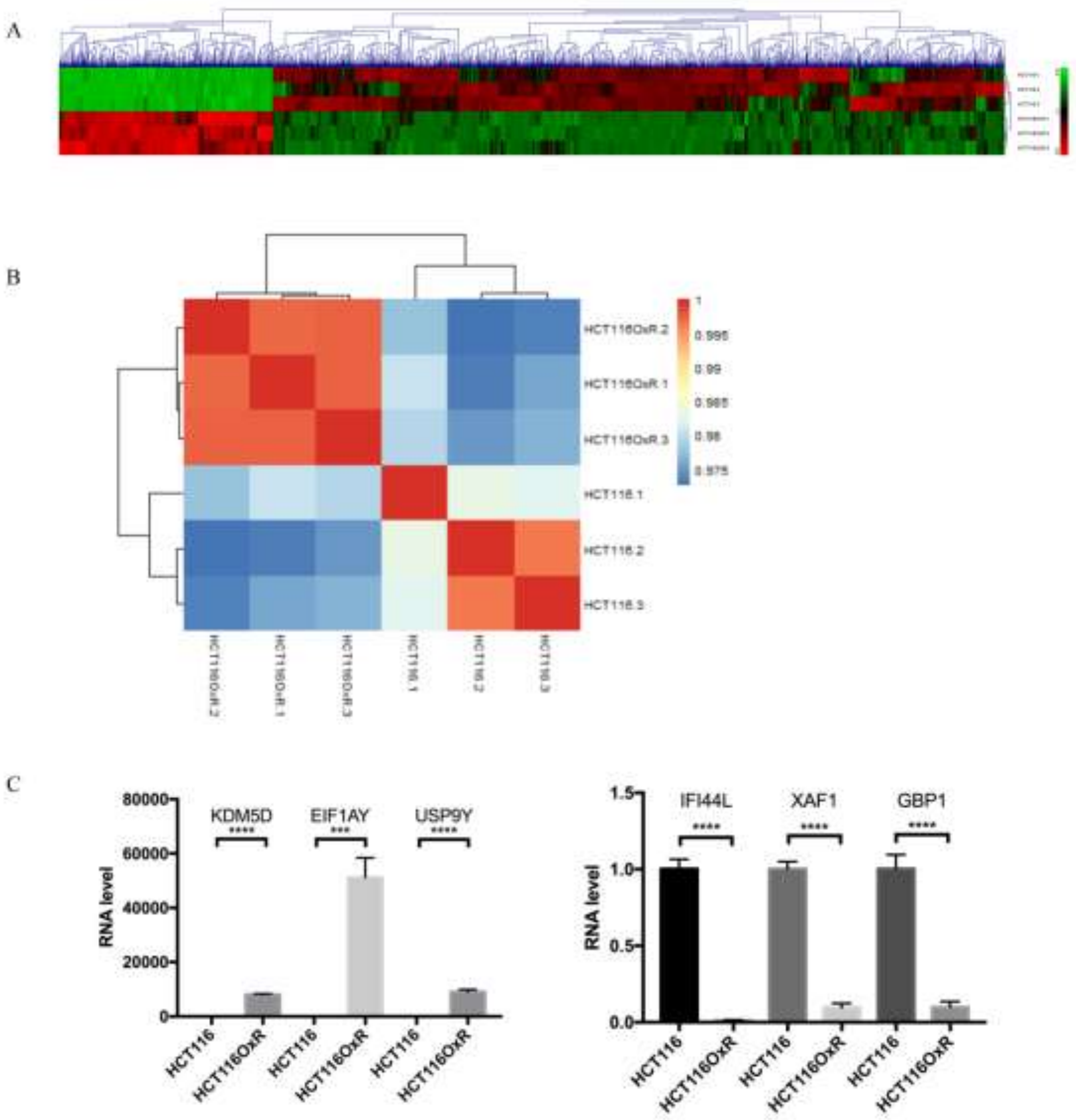

Figure S5. Differential genes detected by RNA-seq.

A Cluster analysis of differentially expressed genes, red indicates high expression genes, green indicates low expression genes. **B** The correlation coefficient heatmap between samples were established by using the identified 32044 transcripts. The results showed that the 3 samples were highly repeatable. **C** qRT-PCR was performed to verify 3 elevated and 3 decreased genes.

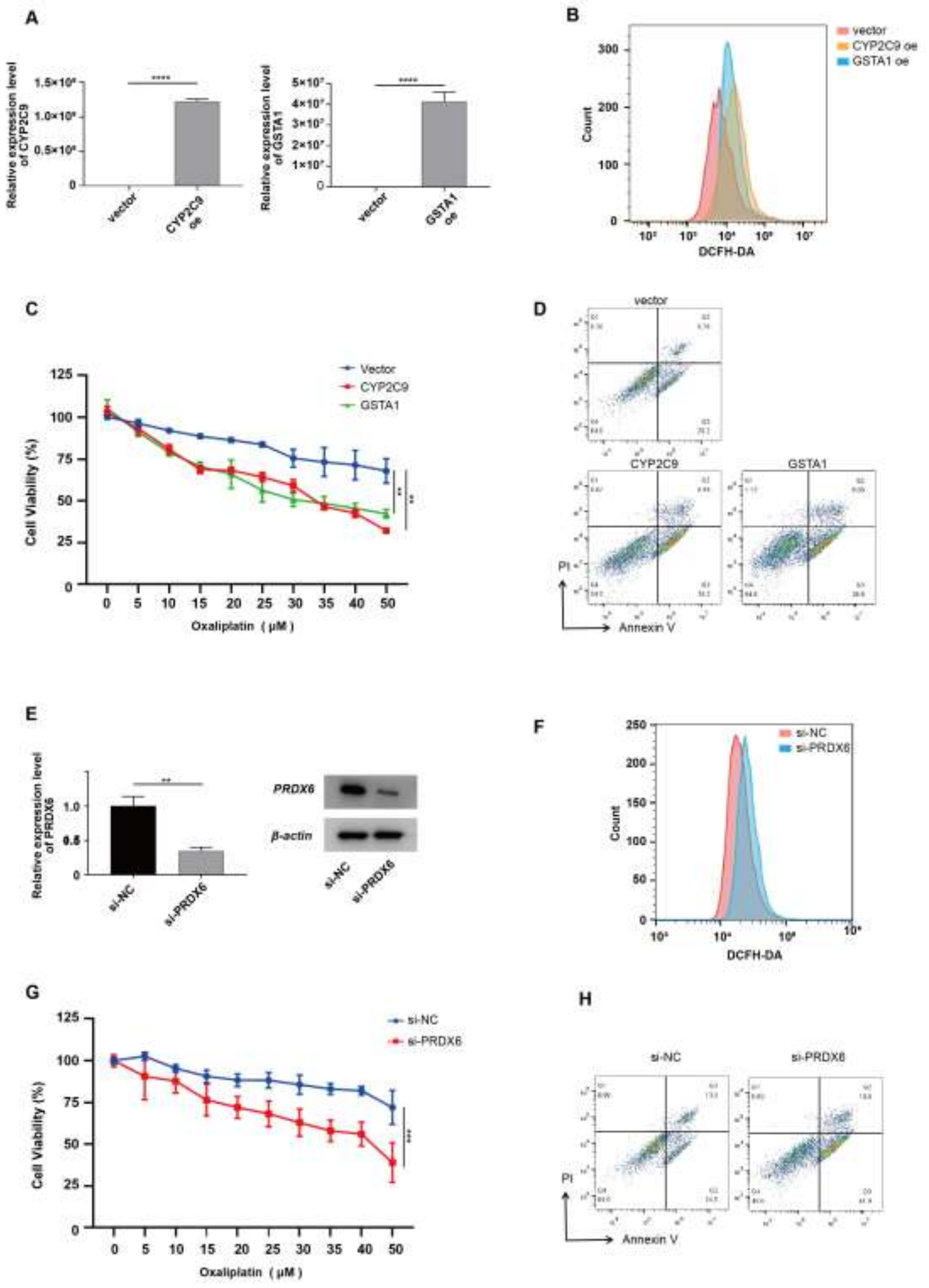

Figure S6. Compartment switching genes can affect cellular response to oxaliplatin by regulating ROS homeostasis.

A The expression of CYP2C9 and GSTA1 mRNA level in HCT116-OxR cells after transduction with indicated overexpression plasmid or vector plasmid was detected by qRT-PCR, ***P<0.001. **B** The level of ROS in indicated cell lines. **C** The responsiveness of HCT116-OxR cells after transduction with indicated overexpression plasmid or vector plasmid to oxaliplatin recorded by CCK8 assay,

P<0.01. **D The percentages of live cells and apoptotic cells was evaluated in indicated cell lines pretreated with oxaliplatin (100 μM, 48 h). **E** The knockdown efficiency of si-PRDX6 in HCT116-OxR was detected by qRT-PCR (left) and Western Blot (right). **F** The level of ROS in HCT116-OxR cells treated with si-PRDX6 or corresponding negative control (si-NC). **G** The responsiveness of HCT116-OxR cells treated with si-PRDX6 or si-NC to oxaliplatin was recorded by CCK8, ***P<0.001. **H** The percentages of live cells and apoptotic cells was evaluated in indicated cell lines pretreated with oxaliplatin (100 μM, 48 h).

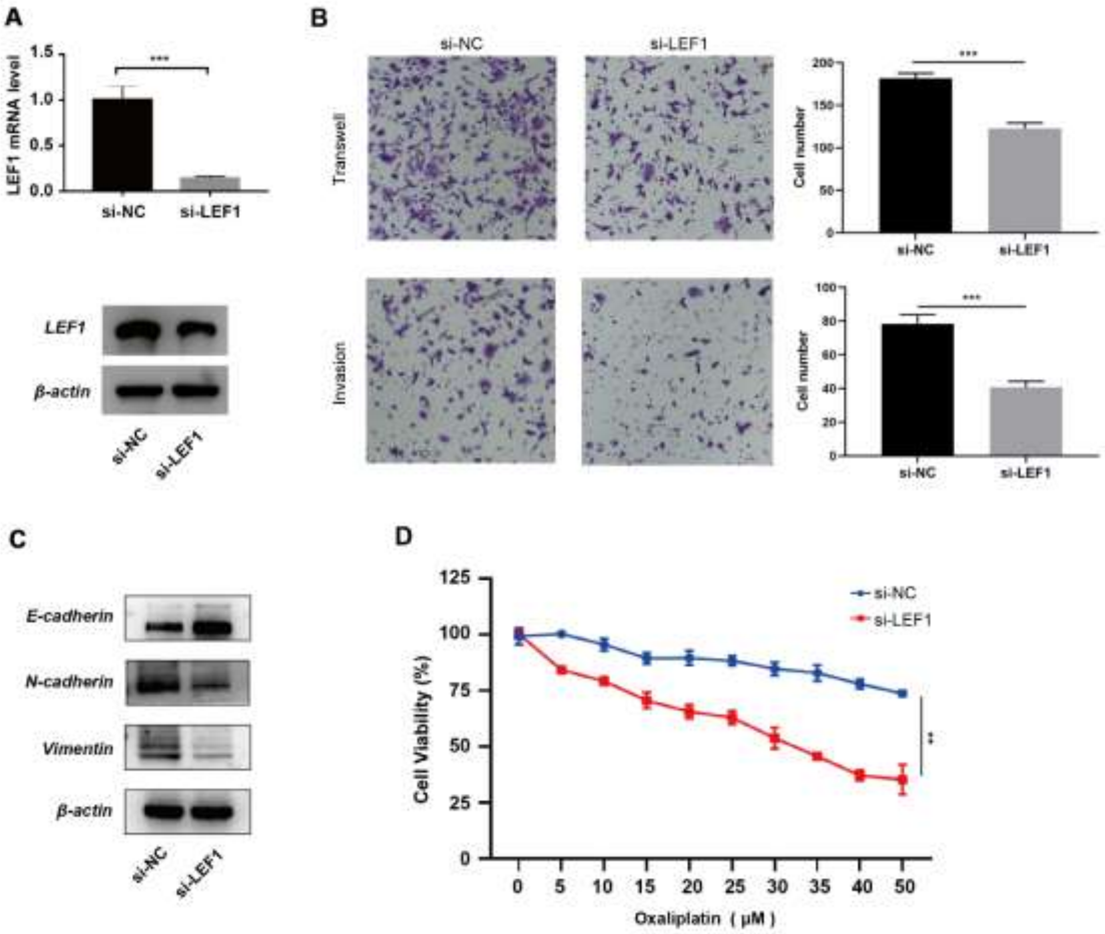

Figure S7. Reducing the expression of LEF1 could weaken the invasion and migration ability of drug-resistant cells.

A The knockdown efficiency of si-LEF1 in HCT116-OxR was detected by qRT-PCR (up) and Western Blot (bottom), ***P<0.001 **B** Cell migration and invasion experiments detected that reducing the expression of LEF1 could reduce the invasion and migration ability of HCT116-OxR cells, **P<0.01. **C** By detecting the expression of EMT markers, it is proved that reducing the expression of LEF1 can reduce the EMT characteristics of drug-resistant cells, ***P<0.001. **D** The responsiveness of HCT116-OxR cells treated with si-LEF1 or si-NC to oxaliplatin was recorded by CCK8, **P<0.01.
